# Association Between Psychological Distress and Incident Dementia in a Population-Based Cohort in Finland

**DOI:** 10.1001/jamanetworkopen.2022.47115

**Published:** 2022-12-15

**Authors:** Sonja Sulkava, Jari Haukka, Raimo Sulkava, Tiina Laatikainen, Tiina Paunio

**Affiliations:** 1 Department of Public Health and Welfare, Finnish Institute for Health and Welfare, Helsinki, Finland; 2 Department of Psychiatry and SleepWell Research Programme, Faculty of Medicine, University of Helsinki and Helsinki University Hospital, Helsinki, Finland; 3 Department of Clinical Genetics, Helsinki University Hospital, Helsinki, Finland; 4 Department of Public Health, University of Helsinki, Helsinki, Finland; 5 Institute of Public Health and Clinical Nutrition, University of Eastern Finland, Kuopio, Finland; 6 Amia Memory Clinics, Helsinki, Finland; 7 Joint Municipal Authority for North Karelia Social and Health Services (Siun Sote), Joensuu, Finland

## Abstract

**Importance:**

Symptoms of psychological distress have shown association with subsequent dementia, but the nature of association remains unclear.

**Objective:**

To examine the association of psychological distress with etiological risk of dementia and incidence of dementia in presence of competing risk of death.

**Design, Setting, and Participants:**

This cohort study consisted of population-based cross-sectional National FINRISK Study surveys collected in 1972, 1977, 1982, 1987, 1992, 1997, 2002, and 2007 in Finland with register-based follow-up; and the cohort was linked to Finnish Health Register data for dementia and mortality for each participant until December 31, 2017. Participants included individuals without dementia who had complete exposure data. Data were analyzed from May 2019 to April 2022.

**Exposures:**

Self-reported symptoms of psychological distress: stress (more than other people), depressive mood, exhaustion, and nervousness (often, sometimes, never).

**Main Outcomes and Measures:**

Incident all-cause dementia, ascertained through linkage to national health registers. Poisson cause-specific hazard model (emphasizing etiological risk) and Fine–Gray subdistribution hazard model (emphasizing effect on incidence) considering dementia and death without dementia as competing risks. Covariates of age, sex, baseline year, follow-up time, educational level, body mass index, smoking, diabetes, systolic blood pressure, cholesterol, and physical activity. Sensitivity analysis was performed to reduce reverse causation bias by excluding individuals with follow-up less than 10 years.

**Results:**

Among 67 688 participants (34 968 [51.7%] women; age range, 25 to 74 years; mean [SD] age, 45.4 years), 7935 received a diagnosis of dementia over a mean follow-up of 25.4 years (range, 10 to 45 years). Psychological distress was significantly associated with all-cause dementia in a multivariable Poisson model, with incidence rate ratios from 1.17 (95% CI, 1.08-1.26) for exhaustion to 1.24 (95% CI, 1.11-1.38) for stress, and remained significant in sensitivity analyses. A Fine–Gray model showed significant associations (with hazard ratios from 1.08 [95% CI, 1.01-1.17] for exhaustion to 1.12 [95% CI, 1.00-1.25] for stress) for symptoms other than depressive mood (hazard ratio, 1.08 [95% CI, 0.98-1.20]). All the symptoms showed significant associations with competing risk of death in both models.

**Conclusions and Relevance:**

In this cohort study, psychological distress symptoms were significantly associated with increased risk of all-cause dementia in the model emphasizing etiological risk. Associations with real incidence of dementia were diminished by the competing risk of death.

## Key Points

**Question:**

What is the association of psychological distress with dementia?

**Findings:**

In this cohort study of 67 688 individuals and, on average, 25 years of follow-up, symptoms of psychological distress—stress, depressive mood, nervousness, and exhaustion—were associated with a 17% to 24% increased risk of dementia in an etiological Poisson model and with an 8% to 12% increase in the incidence of dementia in the Fine-Gray model.

**Meaning:**

These findings suggest that psychological distress is likely to be an etiological risk factor for dementia and associated with the incidence of dementia.

## Teaser

This cohort study examines the association of psychological distress with etiological risk of dementia and incidence of dementia in presence of competing risk of death in a population-based cohort in Finland.

## Introduction

Epidemiological risk factor studies on dementia have a couple of specific features. First, dementia disorders such as Alzheimer disease (AD) have a long preclinical period when underlying neuropathology often already manifests some prodromal symptoms.^1^ Studies with an aged population and short follow-up thus fail to separate prodromal symptoms of dementia from causal risk factors. Second, occurrence of death precludes the occurrence of dementia. The competing risk of death is a source of survival bias in dementia studies and should be accounted for and reported in epidemiological studies of dementia.^2^

Psychological distress refers to nonspecific symptoms of anxiety, depression, perceived stress, and somatic complaints not severe enough to fulfill the diagnostic criteria for psychiatric illness.^3^ Psychological distress as well as clinically important depression and anxiety have shown association with subsequent dementia.^4,5,6,7,8^ However, it is the subject of debate as to whether they are prodromal symptoms or causal risk factors for dementia.^4,9,10,11^

Most of the studies on psychological distress and dementia risk have a limited number of dementia cases, and to our knowledge none of them have modeled the competing risk of death.^5,6,8,12,13^ The objective of this study was to use a large data set (n = 67 688) from the National FINRISK Study surveys with up to a 45-year follow-up to examine the association of symptoms of psychological distress (perceived stress, depressive mood, nervousness, and exhaustion) with subsequent dementia (n = 7935) in models accounting for competing risk of death.

## Methods

### Study Design and Population

This prospective study used data from the National FINRISK Study, a Finnish health and risk factor study consisting of independent cross-sectional cohorts collected every 5 years from 1972 onward (FINRISK surveys 1972, 1977, 1982, 1987, 1992, 1997, 2002, and 2007 included here). Each survey is a combination of random samples of permanent inhabitants aged 25 years to 65 or 75 years representing selected regions of Finland.^14^

The FINRISK surveys 1972 to 2007 had altogether 89 259 invitees, and our total sample included 69 254 (77.6%) of them. The participation rate has varied over the years with a gradually declining trend.^14^ There is an overrepresentation of male, less educated, and younger individuals among the nonparticipants^14^ who also had worse health behavior and higher mortality during the follow-up.^15,16^ This study followed the Strengthening the Reporting of Observational Studies in Epidemiology (STROBE) reporting guideline for cohort studies.

### Ethical Review of Study and Informed Consent

The FINRISK surveys 1992-2007 obtained permissions from the ethical committee of the Finnish Institute of Health and Welfare and/or the Coordinating Ethical Committee of Helsinki and Uusimaa Hospital District. Before the FINRISK survey 1992 ethic committee assessment was not required in Finland, but declaration of Helsinki was followed and confidentiality, anonymity, and data protection have been assured according to Finnish legislation. Informed written consents were obtained from 1997 onward.

### Exposures

Stress was assessed with the question “‘Have you felt yourself tense, stressed or under a lot of strain during the past month?’ 1 = yes, my life is almost unbearable, 2 = yes, quite more so than people usually are, 3 = yes, somewhat, but no more than what is usual, 4 = not at all.” The variable was dichotomized by grouping together 1 and 2 (more than other people) and 3 and 4 (at the same level as other people).

Depressive mood, nervousness, and exhaustion were questioned as follows: “Think of the past month. Please mark the alternative which best describes how often the asked thing or symptom has been on your mind. Do you feel depressed? Do you feel tense and nervous? Do you feel exhausted and overworked?” The answer options were 1 = often, 2 = sometimes, and 3 = not at all. The scales were reversed for the analyses.

### Covariates

The covariates in the basic model included self-reported sex, FINRISK survey year, follow-up time (10-year time slots, Poisson model), age at the end of the follow-up (5-year time slots, Poisson model), and educational classes, which were sex-specific and birth cohort–specific tertiles of schooling years. The fully adjusted model was additionally adjusted for other available established dementia risk factors^11^ that have shown association with psychological distress: systolic blood pressure (average of 1 to 3 measurements), body mass index, total cholesterol, smoking (nonsmoker, current smoker, or ex-smoker), physical activity (combined occupational, leisure, and commuting activity as low, moderate, or high^17,18^), and self-reported diabetes. Insomnia symptoms (often, sometimes, not at all) and alcohol consumption at the level that increases dementia risk^19^ (0 to 14 units in the last week; greater than 14 units in the last week) served as additional covariates for secondary analyses. Complete data set was used for each analysis.

### Follow-up and Outcomes

Follow-up of the participants in each FINRISK survey continued until death, the earliest register-based information of the diagnosis of dementia, or the end of year 2017 (censoring), whichever occurred first. The maximum follow-up time for each survey ranged from 10 to 45 years. We excluded individuals with prevalent dementia at baseline (n = 26).

The diagnoses of all-cause dementia, secondary outcome AD, and death were defined based on combined information from the Finnish national registers: the Hospital Discharge Register and the Causes of Death Register, the Drug Reimbursement Register (reimbursement for AD medication), and the Social Insurance Institution’s information from prescribed and bought dementia drugs (eMethods and eTable 1 in Supplement 1).

### Statistical Analysis

Incidence of all-cause dementia and death without dementia diagnosis were treated as competing risks.^20^ We studied in parallel 2 different hazard regression models for dementia accounting for competing risks: a cause-specific hazard model, here the Poisson cause-specific hazard model, which emphasizes etiological risk factors, and a subdistribution hazard model, here the Fine–Gray subdistribution hazard model, which roughly estimates the effect on cumulative incidence.^21,22^

The 95% CIs and significance threshold of *P* < .05 (Poisson and Fine-Gray models, Pearson χ^2^ and 1-way analysis of variance in cross-sectional secondary analyses) were used throughout the study, except for Spearman correlation for examination of the correlation of the study traits, which used a significance threshold of *P* < .01. Testing was 2-sided.

To reduce reverse causation bias due to the prodromal phase of dementia disorders, we performed sensitivity analyses excluding individuals with short (less than 10-year) follow-up. We performed several secondary analyses: the association of age at exposure was examined by analyses stratified by baseline age (less than 45 years; 45 to 65 years; greater than 65 years) concordant with the age groups in the report of the *Lancet* Commission.^11^ The interaction term with sex in the Poisson model was examined, and if significant, the sex-stratified analyses were performed. As a secondary analysis, we also adjusted our main analyses for insomnia and alcohol consumption. The secondary analyses including analysis of secondary outcome AD should be considered as exploratory because of the potential for inflated type 1 error.

Data were analyzed using R software version 4.0.0 or 4.0.2 (figures) (R Project for Statistical Computing) with packages Epi, cmprsk, and survival. For exploratory cross-sectional analyses, SPSS Statistics version 28.0.0.0 (IBM) was used. Statistical analysis was performed May 2019 to April 2022.

## Results

Of our total sample of 69 254 participants, 67 688 (34 968 [51.7%] women; age range, 25 to 74 years; mean [SD] baseline age, 45.2 [12.2] years) had no prevalent dementia and nonmissing covariates for the basic model (Table 1); the corresponding number was 59 707 for the fully adjusted model (see Table 2 for trait-specific numbers). Characteristics of the individuals with missing covariates are presented in eTable 2 in Supplement 1.

**Table 1.** Baseline Characteristics According to Dementia and Competing Event Status at the End of Follow-up in the Sample With Nonmissing Covariates of the Basic Model

Participant characteristics	FINRISK study surveys	No./total No. (%)		
		All	Dementia	Competing event, death without dementia
Individuals with nonmissing covariates of the basic model (sex, age, educational class, cohort)		67 688	7935	19 647
Exposures				
Stress, more than other people	1982-2002	5670/37 314 (15.2)	417/3376 (12.4)	1120/7877 (14.2)
Depressive mood, often	1972-2002	3393/60 069 (5.6)	502/7469 (6.7)	1380/18 656 (7.4)
Nervousness, often	1972-2002	4872/60 405 (8.1)	720/7545 (9.5)	1957/18 703 (10.5)
Exhaustion, often	1972-2007	8688/66 286 (13.1)	1227/7704 (15.9)	3223/19 071 (16.9)
Covariates				
Female sex	1972-2007	34 968/67 688 (51.7)	4851/7935 (61.1)	7790/19 647 (39.6)
Baseline age, mean (SD), y	1972-2007	45.2 (12.2)	52.5 (9.9)	50.2 (10.7)
Age at the end of follow-up or age at event (dementia/death), mean (SD), y	1972-2007	69.9 (12.2)	79.0 (7.4)	70.9 (11.9)
Educational class, high	1972-2007	24 927/67 688 (36.8)	2931/7935 (36.9)	6921/19 647 (35.2)
Total cholesterol, mean (SD), mmol/l	2002-2007	6.0 (1.3)	6.5 (1.2)	6.5 (1.4)
Body mass index, mean (SD), kg/m^2^	2002-2007	26.4 (4.4)	27.1 (4.1)	27.3 (4.6)
Systolic blood pressure, mean (SD), mmHg	2002-2007	139.5 (20.9)	146.4 (20.9)	148.9 (22.4)
Self-reported diabetes	2002-2007	1850/65 387 (2.8)	254/7799 (3.3)	964/19 353 (4.9)
Physical activity, low	2002-2007	5416/63 403 (8.5)	707/7099 (10.0)	2464/17 798 (13.8)
Smoking, current	2002-2007	17 952/66 771 (26.9)	1260/7748 (16.3)	7122/19 194 (37.1)

**Table 2.** Associations of Psychological Distress Symptoms With All-Cause Dementia in the Cause-Specific Hazard Model (Poisson) and Subdistribution Hazard Model (Fine-Gray)

Trait	No., basic^a^/fully adjusted^b^ model			All-cause dementia				Competing risk of death	
				Cause-specific hazard model, IRR (95% CI)		Fine-Gray subdistribution hazard model, HR (95% CI)		Fully adjusted cause-specific hazard model, IRR (95% CI)^b^	Fully adjusted Fine-Gray subdistribution hazard model, HR (95% CI)^b^
	All	Dementia cases	Death, competing event	Basic^a^	Fully adjusted^b^	Basic^a^	Fully adjusted^b^		
**Stress**									
At same level as other people	31 644/29 014	2959/2516	6757/5865	1 [Reference]	1 [Reference]	1 [Reference]	1 [Reference]	1 [Reference]	1 [Reference]
More than other people	5670/5278	417/367	1120/982	1.23 (1.11-1.37)	1.24 (1.11-1.38)	1.06 (0.95-1.17)^c^	1.12 (1.00-1.25)^c^	1.15 (1.10-1.21)	1.13 (1.05-1.21)^c^
**Depressive mood**									
Never	31 031/28 262	3618/3165	9231/8071	1 [Reference]	1 [Reference]	1 [Reference]	1 [Reference]	1 [Reference]	1 [Reference]
Sometimes	25 645/23 393	3349/2929	8045/7029	1.12 (1.07-1.18)	1.09 (1.04-1.15)	1.05 (0.998-1.09)^c^	1.06 (1.01-1.11)^c^	1.06 (1.04-1.08)	1.08 (1.05-1.12)^c^
Often	3393/2964	502/420	1380/1164	1.28 (1.17-1.41)	1.22 (1.10-1.36)	1.03 (0.94-1.13)^c^	1.08 (0.98-1.20)^c^	1.32 (1.27-1.38)	1.32 (1.24-1.41)^c^
**Nervousness**									
Never	20 897/18 909	2546/2207	6563/5697	1 [Reference]	1 [Reference]	1 [Reference]	1 [Reference]	1 [Reference]	1 [Reference]
Sometimes	34 636/31 677	4279/3745	10 283/9005	1.07 (1.02-1.12)	1.05 (1.00-1.11)	1.05 (1.00-1.10)^c^	1.06 (1.00-1.11)^c^	0.96 (0.94-0.98)	1.01 (0.98-1.05)^c^
Often	4872/4290	720/615	1957/1670	1.24 (1.14-1.35)	1.21 (1.11-1.33)	1.06 (0.98-1.15)^c^	1.11 (1.02-1.22)^c^	1.16 (1.11-1.21)	1.24 (1.17-1.31)^c^
**Exhaustion**									
Never	21 029/18 781	2348/2013	5975/5193	1 [Reference]	1 [Reference]	1 [Reference]	1 [Reference]	1 [Reference]	1 [Reference]
Sometimes	36 569/32 578	4129/3614	9873/8620	1.07 (1.02-1.13)	1.08 (1.02-1.14)	1.05 (0.99-1.10)^d^	1.06 (1.01-1.12)	1.02 (0.99-1.04)	1.01 (0.98-1.05)
Often	8688/7573	1227/1047	3223/2742	1.20 (1.12-1.29)	1.17 (1.08-1.26)	1.04 (0.97-1.12)^d^	1.08 (1.01-1.17)	1.18 (1.15-1.23)	1.13 (1.08-1.19)

Abbreviations: HR, hazard ratio; IRR, incidence rate ratio.

^a^ Basic model adjusted for follow-up time (10-year time slots, Poisson model), age at the end of follow-up (5-year time slots, Poisson model), FINRISK survey year, sex, and educational class.

^b^ Fully adjusted model adjusted for FINRISK survey year, follow-up time (10-year time slots, Poisson model), age at the end of follow-up (5-year time slots, Poisson model), sex, educational class, body mass index, systolic blood pressure, total cholesterol, smoking, physical activity, and diabetes.

^c^ No covariate of FINRISK survey year could be included.

^d^ Education coded as years of education for numerical reasons.

Table 1 provides baseline descriptive statistics for the study sample. The competing risk of death was more common than dementia (19 647 vs 7935). The mean (SD) age of death without dementia was 70.9 (11.9) years and for onset of dementia was 79.0 (7.4) years. The Figure visualizes increased cumulative incidence of competing events for individuals with “often” depressive mood, exhaustion, nervousness, or stress from the age of 45 to 55 years onward. The symptomatic groups also show slightly increased cumulative incidence of dementia between 70 and 90 years of age and slightly decreased cumulative incidence of dementia after the age of 90 years. The different symptoms of psychological distress correlated significantly in the cross-sectional data (Spearman ρ, 0.36 to 0.52) (eTable 3 in Supplement 1).

**Figure.**
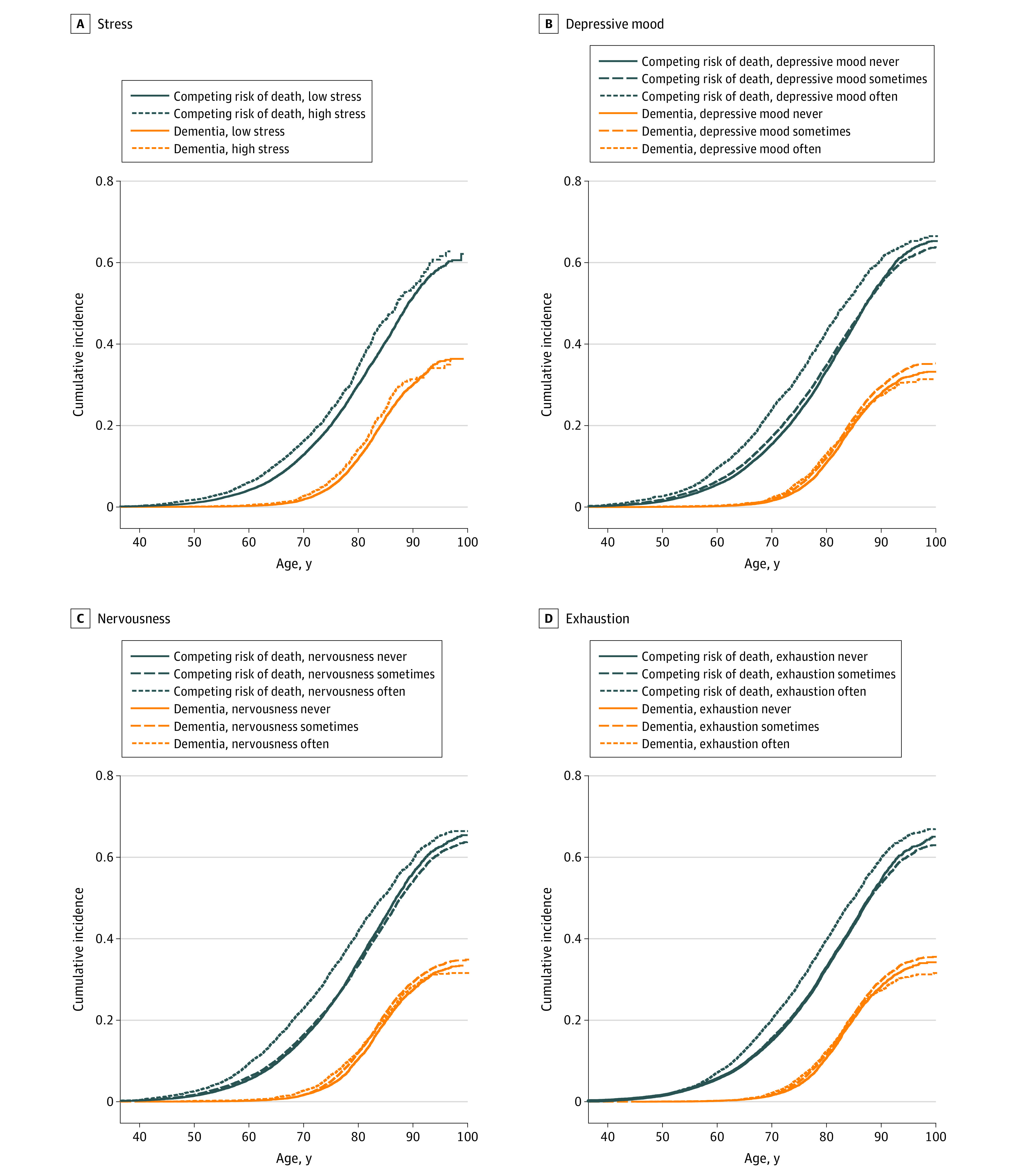
Cumulative Incidence of All-Cause Dementia and Competing Risk of Death for Symptoms of Psychological Distress

### Hazard Models for All-Cause Dementia and Competing Risk of Death

We fit the Poisson cause-specific model (Poisson model, emphasizing etiology) and the Fine–Gray subdistribution hazard model (Fine–Gray model, reflecting incidence of dementia over time) for all-cause dementia and competing risk of death. For both models we regressed the hazard separately on symptoms of psychological distress (stress, depressive mood, exhaustion, and nervousness) and other covariates.

In the Poisson model, the symptoms of psychological distress “often” were associated with an increase in the cause-specific hazard of dementia with incidence rate ratios (IRRs) from 1.20 (95% CI, 1.12-1.29) for exhaustion to 1.28 (95% CI, 1.17-1.41) for depressive mood (Table 2). The associations remained significant in the fully adjusted model. For depressive mood, nervousness, and exhaustion symptoms reported with a frequency scale (“never,” “sometimes,” “often”) a dose–response effect was visible. The associations remained statistically significant after exclusion of individuals with less than 10 years follow-up (eTable 4 in Supplement 1).

In the Fine-Gray analyses, not in the basic model but in the fully adjusted model, stress (hazard ratio [HR], 1.12 [95% CI, 1.00-1.25]), nervousness (HR, 1.11 [95% CI, 1.02-1.22]), and exhaustion (HR, 1.08 [95% CI, 1.01-1.17]) showed significant associations, but depressive mood did not (HR, 1.08 [95% CI, 0.98-1.20]). All the symptoms were associated with competing risk of death in both the Poisson and Fine–Gray models (Table 2).

The individuals with missing covariates in the fully adjusted model showed association with all-cause dementia (IRR, 1.09 [95% CI, 1.02-1.16]) and competing risk of death (IRR, 1.20 [95% CI, 1.17-1.23]) in the basic model (Poisson) (eTable 5 in Supplement 1). When considering the individuals with missing data in the basic model, in the analyses including the nonrespondents as a separate category the association of psychological distress symptoms and dementia was unchanged (eTable 6 in Supplement 1).

The associations of depressive mood and exhaustion with dementia in Poisson model showed significant sex differences (interaction term IRR, 0.73 [95% CI, 0.58-0.91] and 0.90 [0.72-1.12]). The significant associations were detected only among male participants (male participants, depressed “often”: IRR, 1.52 [95% CI, 1.26-1.84], and exhaustion “often”: IRR, 1.32 [95% CI, 1.16-1.51]; female participants, depressed “often”: IRR, 1.12 [95% CI, 0.99-1.27], and exhaustion “often”: 1.08 [95% CI, 0.98-1.19]) (eTable 7 in Supplement 1).

As secondary analyses we studied separately the association of the psychological distress symptoms with dementia in the baseline age groups of early life (<45 years), midlife (45-65 years), and late life (>65 years) (Table 3). The associations from the Poisson model were significant in all the subgroups for all the symptoms “often,” except for nervousness in late life and stress in early life. As a secondary analysis, we also adjusted our Poisson analyses for insomnia, which resulted in smaller but significant effect sizes (eTable 8 in Supplement 1), and alcohol consumption, which only marginally affected the results (eTable 9 in Supplement 1).

**Table 3.** Associations of Psychological Distress Symptoms With All-Cause Dementia in Baseline Age Groups in Fully Adjusted Cause-Specific Hazard Model (Poisson)^a^

Trait	Early life (<45 y)			Midlife (45-65 y)			Late life (>65 y)		
	No.		IRR (95% CI)	No.		IRR (95% CI)	No.		IRR (95% CI)
	All	Dementia		All	Dementia		All	Dementia	
**Stress**									
At the same level as other people	14 224	218	1 [Reference]	13 515	4165	1 [Reference]	1275	334	1 [Reference]
More than other people	2836	40	1.15 (0.82-1.61)	2328	666	1.21 (1.07-1.38)	114	38	1.49 (1.06-2.10)
**Depressive mood**									
Never	14 884	769	1 [Reference]	12 458	5156	1 [Reference]	920	224	1 [Reference]
Sometimes	12 759	801	1.09 (0.98-1.20)	10 223	4551	1.08 (1.01-1.15)	411	126	1.36 (1.09-1.71)
Often	1345	102	1.32 (1.07-1.63)	1573	814	1.15 (1.02-1.31)	46	18	2.31 (1.40-3.80)
**Nervousness**									
Never	9267	483	1 [Reference]	8903	3766	1 [Reference]	739	191	1 [Reference]
Sometimes	17 645	1019	0.97 (0.87-1.08)	13 434	5732	1.08 (1.02-1.16)	598	165	1.12 (0.90-1.38)
Often	2175	180	1.24 (1.04-1.48)	2070	1101	1.19 (1.07-1.33)	45	14	1.47 (0.85-2.54)
**Exhaustion**									
Never	9767	496	1 [Reference]	8016	3154	1 [Reference]	998	183	1 [Reference]
Sometimes	17 318	944	1.02 (0.92-1.14)	14 146	5466	1.06 (0.99-1.13)	1114	235	1.52 (1.24-1.85)
Often	3219	240	1.18 (1.00-1.38)	4190	2048	1.12 (1.02-1.22)	164	45	1.74 (1.23-2.48)

Abbreviation: IRR, incidence rate ratio.

^a^ Fully adjusted model adjusted for FINRISK survey year, follow-up time (10-year time slots), age at the end of follow-up (5-year time slots), sex, educational class, body mass index, systolic blood pressure, total cholesterol, smoking, physical activity, and diabetes.

### Hazard Models for Alzheimer Disease

As a secondary outcome we studied AD, which composed 73.9% (5865 of 7935) of all dementia cases. In the Poisson model for AD, nervousness (IRR, 1.14 [95% CI, 1.03-1.26]) and exhaustion (IRR, 1.10 [95% CI, 1.01-1.20]) “often” showed significant association in the basic model and fully adjusted models (Table 4). Depressive mood “often” did not show significant association with AD, and stress showed significant association only in the fully adjusted model (IRR, 1.24 [95% CI, 1.11-1.38]).

**Table 4.** Associations of Psychological Distress Symptoms With Alzheimer Disease in the Cause-Specific Hazard Model (Poisson) and Subdistribution Hazard Model (Fine-Gray)

Trait	No., basic^a^/fully adjusted^b^ model		Cause-specific hazard model, IRR (95% CI)		Fine-Gray subdistribution hazard, HR (95% CI)	
	All	AD cases	Basic model^a^	Fully adjusted model^b^	Basic model^a^	Fully adjusted model^b^
Stress						
At the same level as other people	31 644/29 014	2356/2023	1 [Reference]	1 [Reference]	1 [Reference]	1 [Reference]
More than other people	5670/5278	299/271	1.09 (0.96-1.23)	1.24 (1.11-1.38)	0.94 (0.83-1.06)^c^	1.02 (0.90-1.16)^c^
**Depressive mood**						
Never	31 031/28 262	2726/2417	1 [Reference]	1 [Reference]	1 [Reference]	1 [Reference]
Sometimes	25 645/23 393	2445/2153	1.08 (1.02-1.14)	1.06 (0.99-1.11)	1.00 (0.95-1.06)^d^	1.001 (0.94-1.06)^c^
Often	3393/2964	326/276	1.12 (0.997-1.26)	1.09 (0.96-1.23)	0.88 (0.79-0.99)^d^	0.94 (0.83-1.06)^c^
**Nervousness**						
Never	20 897/18 909	1872/1643	1 [Reference]	1 [Reference]	1 [Reference]	1 [Reference]
Sometimes	34 636/31 677	3198/2826	1.08 (1.02-1.14)	1.07 (1.004-1.14)	1.06 (1.00-1.12)^d^	1.06 (0.997-1.12)^c^
Often	4872/4290	483/416	1.14 (1.03-1.26)	1.13 (1.01-1.26)	0.97 (0.88-1.07)^d^	1.01 (0.91-1.13)^c^
**Exhaustion**						
Never	21 029/18 781	1805/1558	1 [Reference]	1 [Reference]	1 [Reference]	1 [Reference]
Sometimes	36 569/32 578	3056/2697	1.03 (0.98-1.10)	1.04 (0.98-1.11)	1.02 (0.96-1.08)^e^	1.02 (0.96-1.09)
Often	3688/7573	829/726	1.10 (1.01-1.20)	1.11 (1.01-1.21)	0.97 (0.89-1.05)^e^	1.01 (0.92-1.10)

Abbreviations: AD, Alzheimer disease; HR, hazard ratio; IRR, incidence rate ratio.

^a^ Basic model adjusted for follow-up time (10-year time slots, Poisson model), age at the end of follow-up (5-year time slots, Poisson model), FINRISK survey year, sex, and educational class.

^b^ Fully adjusted model adjusted for FINRISK survey year, follow-up time (10-year time slots, Poisson model), age at the end of follow-up (5-year time slots, Poisson model), sex, educational class, body mass index, systolic blood pressure, total cholesterol, smoking, physical activity, and diabetes.

^c^ No covariate of FINRISK survey year could be included.

^d^ Education coded as years of education for numerical reasons.

^e^ Education coded as years of education and covariate of smoking included for numerical reasons.

In the Fine–Gray model, the symptoms of psychological distress did not show association with incidence of AD except for depressive mood “often,” which showed association with decreased relative incidence of AD in the basic model (HR, 0.88 [95% CI, 0.79-0.99]), which, however, was not significant in the fully adjusted model (HR, 0.94 [95% CI, 0.83-1.06]) (Table 3). The sensitivity analyses excluding individuals with less than 10 years follow-up showed no significant associations (eTable 10 in Supplement 1).

## Discussion

This cohort study found a higher risk of all-cause dementia associated with psychological distress manifesting as symptoms of stress, depressive mood, nervousness, or exhaustion. The association was not due to confounding cardiovascular risk factors or reverse causation of prodromal dementia disorder. In addition, the presence of the competing risk of death was demonstrated, and it likely weakens the effect of psychological distress on the real incidence of dementia.

Overall, the increase in the dementia risk (hazard) associated with the psychological distress symptoms in our study was around 20% in the Poisson model, and it was not fully caused by reverse causation. As assumed, the effect sizes were smaller than those reported for psychiatric disorders,^23,24,25^ which are likely to represent more severe situations. Our results agree with previous longitudinal studies reporting multiple symptoms of psychological distress or an aggregate of the different symptoms associated with modestly increased risk of dementia in etiological models.^5,6,8,12,13^ Most of the studies have included a limited number of cases (less than 500) and none of the studies, to our knowledge, have analyzed competing risk of death and effect on the incidence of dementia, however. Thus, in addition to confirmation of the association in the etiological model, we report here an association of psychological distress on the real incidence of dementia in the presence of the possibility of death from other causes (Figure, Table 2)

Of the separate symptoms, perceived stress was associated with an increased risk of dementia in a few previous studies with a low number of cases^7,26^ and in a small meta-analysis with 203 cases.^27^ Thus, our results support that association with a larger data set. Psychological underpinnings of nervousness and stress are closely related to those of anxiety and may reflect effects of the same coping style. Meta-analysis of anxiety, mostly based on scales or questionnaires, showed marginally significant association with 45% increase in the Alzheimer disease risk.^28^ The median follow-up time being 9 years, reverse-causation may have led to higher Alzheimer disease risk as compared with our results on nervousness and stress (Table 3), which were not significant after excluding individuals with follow-up time less than 10 years.

Several previous studies have indicated that depressive symptoms in late life, but not in midlife, increase dementia risk, suggesting depression is a prodromal feature and not a causal risk factor for dementia.^10,29,30^ In contrast, in our large study, the association of the 1-item question on depressive mood remained significant in the sensitivity analyses for reverse causation and in the subgroup analyses studying depressive mood in early life and midlife.

Interestingly, associations of depressive mood and exhaustion with dementia were detected significantly only in men (eTable 7 in Supplement 1), as reported previously in.^31^ This could indicate that among men reporting depressive symptoms “often,” the degree of symptoms may be more severe and thereby relate more strongly to an increased risk of dementia.

All the symptoms showed a weaker effect size in the Fine–Gray model, reflecting the association with the true incidence, compared with the Poisson model, which emphasizes etiological risk (Table 2). Selective survival is the likely explanation for the difference, because individuals with psychological distress have an increased risk of dying before getting dementia (Table 2), due to, for example, cardiovascular and cancer diseases.^32^ The effect of competing risk on the incidence but not the cause-specific hazard of the main outcome has been demonstrated before.^20^

In the secondary subgroup analyses, higher IRRs in early life symptoms compared with midlife symptoms may indicate that early life symptoms indicate a higher personal tendency toward psychological distress compared with symptoms later, but they may also reflect the existence of selective enrollment bias. If psychological distress together with some other risk factors has more than a multiplicative effect on early mortality, it leads to excess mortality of individuals with psychological distress and other risk factors and thus pre-enrollment selection of individuals with psychological distress having fewer other risk factors. Thus, in the cohort with older baseline age, the association with outcome is underestimated,^2,33^ as demonstrated for smoking and dementia.^34^ In our data, deaths occurred during midlife but rarely during early life (Figure). Thus, in the early life group the risk estimate may be closer to the true effect size due to less selective enrollment bias.

Our study traits correlated significantly (Spearman ρ, 0.36 to 0.52) (eTable 3 in Supplement 1). It is likely that the symptoms partially reflect the same signal of emotional load, which we call by the umbrella term psychological distress. The similar association results do not encourage highlighting of the role of one symptom over another as a risk factor for dementia. The questions may also tag the trait-like individual tendency to react to stress with anxiety and depression, neuroticism, which has shown association with dementia risk and faster cognitive decline in older adults.^35,36^ Anxiety symptoms have also shown association with faster cognitive decline in the presence of amyloid beta pathology.^37^

AD studied as a secondary outcome showed significant and weaker than all-cause dementia associations with psychological distress, which did not survive sensitivity analysis for reverse causation. It is possible that low sensitivity of the registers for AD before the mid-1990s (eMethods in Supplement 1) leads to underestimation of the association, especially in the sensitivity analysis requiring follow-up of more than 10 years. Another explanation would be that the symptoms of psychological distress increase the risk of, for example, vascular dementia, the second most prevalent dementia.^38^ Psychological distress may lead to unhealthy lifestyle behaviors or avoidance of medical screening because of fear of having a serious illness.^39,40^ Indeed, mental health problems have been linked with cardiovascular diseases and even increased atherosclerosis.^41^ However, adjusting for cardiovascular risk factors did not diminish the associations with all-cause dementia (Table 2).

In addition to cardiovascular factors, mechanisms to mediate the associations detected here may include hypothalamic-pituitary-adrenal axis dysfunction,^42,43,44^ neuroinflammation,^45^ and reduced brain-derived neurotrophic factor.^46^ Sleep problems may also be mediators because a lack of slow-wave sleep is suggested to reduce the clearance of amyloid beta and tau from the brain through the glymphatic system,^47,48^ and insomnia symptoms have been suggested to increase the risk of dementia or AD.^49,50^ Controlling for insomnia symptoms, indeed, weakened the associations suggesting sleep problems as a partly mediating mechanism (eTable 9 in Supplement 1).

### Limitations

This study has several limitations. First, information on 3 established risk factors for dementia,^11^ which may associate with psychological distress, was not available: traumatic brain injury, hearing impairment, and low social contact. They or some unknown factor may confound the analyses.

Second, individuals with missing information on the covariates or those who did not participate^15,16^ had more risk factors for, and increased risk of, dementia or mortality (eTable 2 and eTable 5 in Supplement 1). The selective participation and nonresponsiveness may potentially bias the associations. The basic model data set has 2.0% to 2.5% of nonrespondents depending on psychological distress trait (eTable 6 in Supplement 1). Inclucion of the nonrespondents as a separate category in the model did not change the association of psychological distress and dementia (eTable 11 in Supplement 1). The participation rate in the National FINRISK study is at reasonable level when compared internationally.^51^ However, nonparticipation prevents generalizability of the results to Finnish population as discussed earlier.^16,52^

Third, our questions on psychological distress do not form any validated multi-item questionnaire. Therefore, we report in parallel several 1-item measures for different symptoms of psychological distress, which correlate significantly (eTable 11 in Supplement 1) and show quite a robust pattern of association with dementia of similar magnitude. We consider that they together reflect the phenomenon of psychological distress.

## Conclusions

The findings of this large Finnish cohort study with register-based follow-up of up to 45 years suggest that competing risk of death, reverse causation, and ascertainment bias are likely to affect estimation of the association of mental health with dementia risk. After considering these phenomena, we suggest that symptoms of psychological distress are etiological risk factors for dementia but only weakly increase the incidence of dementia in the presence of competing risk of death.

## Notes

Supplement 1. **eTable 1.** Number of Dementia Diagnoses by Finnish Health-Care Registers and the Source for the Earliest Diagnosis of Dementia **eTable 2.** Baseline Characteristics of the Individuals With and Without Missing Data on Covariates of the Fully Adjusted Model and No Prevalent Dementia **eTable 3.** Correlation of Study Traits **eTable 4.** Sensitivity Analyses for Associations of Psychological Distress Symptoms With All-Cause Dementia in the Fully Adjusted Cause-Specific Hazard Model (Poisson) and Subdistribution Hazard Model (Fine–Gray) (Only Individuals With Follow-Up Time ≥ 10 Years Are Included) **eTable 5.** Associations of Missing Data Status With All-Cause Dementia in Basic Cause-Specific Hazard Model (Poisson) and Subdistribution Hazard Model (Fine–Gray) **eTable 6.** Associations of Psychological Distress Symptoms or Nonresponsiveness With All-Cause Dementia in Basic Cause-Specific Hazard Model (Poisson). **eTable 7.** Associations of Depressive Mood and Exhaustion With All-Cause Dementia in Fully Adjusted Cause-Specific Hazard Model (Poisson) Stratified by Sex **eTable 8.** Associations of Psychological Distress Symptoms With All-Cause Dementia in Fully Adjusted Cause-Specific Hazard Model (Poisson) With Covariate Of Insomnia **eTable 9.** Associations of Psychological Distress Symptoms With All-Cause Dementia in Fully Adjusted Cause-Specific Hazard Model (Poisson) With and Without Covariate of Alcohol Consumption in the Cohort Comprising of the FINRISK Study Surveys 1982, 1987 and 1992 **eTable 10.** Sensitivity Analyses For Associations of Psychological Distress Symptoms with Alzheimer’s Disease in the Fully Adjusted Cause-Specific Hazard Model (Poisson) and Subdistribution Hazard Model (Fine–Gray) (Only Individuals With Follow-Up Time ≥ 10 Years Are Included) **eTable 11.** Depressive Mood and Self-Reported Diagnosis of Depression Stratified By Sex in the FINRISK 2002 Survey
eMethods

Supplement 2.
Data Sharing Statement
