# Supplementary material for: Association Between Psychological Distress and Incident Dementia in a Population-Based Cohort in Finland
Source: JAMA Netw Open. 2022 Dec 15;5(12):e2247115. doi: 10.1001/jamanetworkopen.2022.47115 (PMC9856411; doi:10.1001/jamanetworkopen.2022.47115)
Supplement: Supplement 1. — eTable 1. Number of Dementia Diagnoses by Finnish Health-Care Registers and the Source for the Earliest Diagnosis of Dementia eTable 2. Baseline Characteristics of the Individuals With and Without Missing Data on Covariates of the Fully Adjusted Model and No Prevalent Dementia eTable 3. Correlation of Study Traits eTable 4. Sensitivity Analyses for Associations of Psychological Distress Symptoms With All-Cause Dementia in the Fully Adjusted Cause-Specific Hazard Model (Poisson) and Subdistribution Hazard Model (Fine–Gray) (Only Individuals With Follow-Up Time ≥ 10 Years Are Included) eTable 5. Associations of Missing Data Status With All-Cause Dementia in Basic Cause-Specific Hazard Model (Poisson) and Subdistribution Hazard Model (Fine–Gray) eTable 6. Associations of Psychological Distress Symptoms or Nonresponsiveness With All-Cause Dementia in Basic Cause-Specific Hazard Model (Poisson). eTable 7. Associations of Depressive Mood and Exhaustion With All-Cause Dementia in Fully Adjusted Cause-Specific Hazard Model (Poisson) Stratified by Sex eTable 8. Associations of Psychological Distress Symptoms With All-Cause Dementia in Fully Adjusted Cause-Specific Hazard Model (Poisson) With Covariate Of Insomnia eTable 9. Associations of Psychological Distress Symptoms With All-Cause Dementia in Fully Adjusted Cause-Specific Hazard Model (Poisson) With and Without Covariate of Alcohol Consumption in the Cohort Comprising of the FINRISK Study Surveys 1982, 1987 and 1992 eTable 10. Sensitivity Analyses For Associations of Psychological Distress Symptoms with Alzheimer’s Disease in the Fully Adjusted Cause-Specific Hazard Model (Poisson) and Subdistribution Hazard Model (Fine–Gray) (Only Individuals With Follow-Up Time ≥ 10 Years Are Included) eTable 11. Depressive Mood and Self-Reported Diagnosis of Depression Stratified By Sex in the FINRISK 2002 Survey eMethods [file jamanetwopen-e2247115-s001.pdf]

Supplemental online content

Sulkava S, Haukka J, Sulkava R, Laatikainen T, Paunio T. Association between psychological distress and incident dementia in a population-based cohort in Finland. *JAMA Netw Open.* 2022;5(12):e2247115. doi:10.1001/jamanetworkopen.2022.47115

eTable 1. Number of Dementia Diagnoses by Finnish Health-Care Registers and the Source for the Earliest Diagnosis of Dementia

eTable 2. Baseline Characteristics of the Individuals With and Without Missing Data on Covariates of the Fully Adjusted Model and No Prevalent Dementia

eTable 3. Correlation of Study Traits

eTable 4. Sensitivity Analyses for Associations of Psychological Distress Symptoms With All-Cause Dementia in the Fully Adjusted Cause-Specific Hazard Model (Poisson) and Subdistribution Hazard Model (Fine–Gray) (Only Individuals With Follow-Up Time ≥ 10 Years Are Included)

eTable 5. Associations of Missing Data Status With All-Cause Dementia in Basic Cause-Specific Hazard Model (Poisson) and Subdistribution Hazard Model (Fine–Gray)

eTable 6. Associations of Psychological Distress Symptoms or Nonresponsiveness With All-Cause Dementia in Basic Cause-Specific Hazard Model (Poisson).

eTable 7. Associations of Depressive Mood and Exhaustion With All-Cause Dementia in Fully Adjusted Cause-Specific Hazard Model (Poisson) Stratified by Sex

eTable 8. Associations of Psychological Distress Symptoms With All-Cause Dementia in Fully Adjusted Cause-Specific Hazard Model (Poisson) With Covariate Of Insomnia

eTable 9. Associations of Psychological Distress Symptoms With All-Cause Dementia in Fully Adjusted Cause-Specific Hazard Model (Poisson) With and Without Covariate of Alcohol Consumption in the Cohort Comprising of the FINRISK Study Surveys 1982, 1987 and 1992

© 2022 Sulkava S et al. *JAMA Network Open.*

eTable 10. Sensitivity Analyses For Associations of Psychological Distress Symptoms with Alzheimer's Disease in the Fully Adjusted Cause-Specific Hazard Model (Poisson) and Subdistribution Hazard Model (Fine–Gray) (Only Individuals With Follow-Up Time ≥ 10 Years Are Included)

eTable 11. Depressive Mood and Self-Reported Diagnosis of Depression Stratified By Sex in the FINRISK 2002 Survey

eMethods

This supplemental material has been provided by the authors to give readers additional information about their work.

eTable 1. Number of dementia diagnoses by Finnish health-care registers and the source for the earliest diagnosis of dementia.

**Diagnosis of dementia**				
**Trait**	**Hospital Discharge Register, No. (%)**	**Drug Reimbursement Register, No. (%)**	**Prescribed and bought dementia drugs from Social Insurance Institution, No. (%)**	**Causes of Death Register, No. (%)**
Incident dementia, n=8211	5787 (70.4)	4174 (50.8)	5245 (63.9)	3668 (44.7)
Incident AD, n=6070	3729 (61.4)	3600 (59.3)	5245 (86.4)	2362 (38.9)

**Earliest diagnosis of dementia**				
**Trait**	**Hospital Discharge Register, No. (%)**	**Drug Reimbursement Register, No. (%)**	**Prescribed and bought dementia drugs from Social Insurance Institution, No. (%)**	**Causes of Death Register, No. (%)**
Incident dementia, n=8211*	3214 (39.1)	3408 (41.5)	1237 (15.1)	408 (5.0)
Incident AD, n=6070*, **	1410 (23.2)	3387 (55.8)	1222 (20.1)	104 (1.7)

*Some individuals received the diagnosis from several registers on the same day; therefore the sum of the cells is greater than the total number of cases. ** If Alzheimer's disease (AD) was diagnosed at some point, the earliest report of dementia was coded as the first occurrence of AD.

© 2022 Sulkava S et al. *JAMA Network Open.*

eTable 2. Baseline characteristics of the individuals with and without missing data on covariates of the fully adjusted model and no prevalent dementia.

Participant characteristics	Non-missing data on covariates of the fully adjusted model	Missing data on covariates of the fully adjusted model	Difference between groups, *P* value[a]
**No.**	59 707**, No. (%)**	9521**, No. (%)**	
**Exposures**			
Stress, *more than other people*	5278/34 292 (15.4)	482/3870 (12.5)	<0.001
Depressive mood, *often*	2964/54 619 (5.4)	494/6695 (7.4)	<0.001
Nervous, *often*	4290/54 876 (7.8)	684/6785 (10.1)	<0.001
Exhaustion, *often*	7573/58 932 (12.9)	13 295/8659 (15.0)	<0.001
**Covariates**			
Sex, *female*	30 755/59 707 (51.5)	5010/9521 (52.6)	0.044
Educational class *intermediate/high*	22 676/59 707 (38.0)	2252/7984 (28.2)	<0.001
Baseline age, *mean (SD), years*	44.8 (11.9)	48.7 (13.1)	<0.001
Age at the end of follow-up or age at event (dementia/death), *mean (SD), years*	69.9 (12.0)	70.4 (13.7)	<0.001
Total cholesterol, *mean (SD), mmol/l*	6.0 (1.3)	6.1 (1.3)	<0.001
Body mass index, *mean (SD)kg/m²*	26.4 (4.4)	27.2 (4.8)	<0.001
Systolic blood pressure, *mean (SD), mmHg*	139.1 (20.7)	142.5 (22.0)	<0.001
Self-reported diabetes	1588/59 707 (2.7)	307/6959 (4.4)	<0.001
Physical activity, *low*	5052/59 707 (8.5)	490/4740 (10.3)	<0.001
Smoking, *current*	16 183/59 707 (27.1)	2147/8353 (25.7)	<0.001

[a] Pearson's chi-square test for categorical variables and one-way analysis of variance for continuous variables. SD, standard deviation.

© 2022 Sulkava S et al. *JAMA Network Open.*

eTable 3. Correlation of study traits.

	Stress	Depressive mood	Nervousness	Exhaustion
**Stress** Spearman's rho	1.00			
No.	38 162			
**Depressive mood** Spearman's rho	0.37*	1.00		
No.	37 330	61 329		
**Nervousness** Spearman's rho	0.36*	0.52*	1.00	
No.	37 451	61 000	61 676	
**Exhaustion** Spearman's rho	0.36*	0.45*	0.45*	1.00
No.	37 326	60 818	61 110	67 614

* Correlation is significant at the 0.01 level (two-tailed).

© 2022 Sulkava S et al. *JAMA Network Open.*

eTable 4. Sensitivity analyses for associations of psychological distress symptoms with all-cause dementia in the fully adjusted cause-specific hazard model (Poisson) and subdistribution hazard model (Fine–Gray). (Only individuals with follow-up time ≥ 10 years are included.)

			**Poisson cause-specific hazard model**	**Fine–Gray subdistribution hazard model**
**Trait**	No., all	No., dementia	Fully adjusted model, IRR (CI 95%)	Fully adjusted model, HR (CI 95%)
**Stress**				
At the same level as other people	27 292	2277	1 (reference)	1 (reference)
More than other people	4931	311	1.18 (1.04–1.32)	1.09 (0.97–1.23)[a]
**Work-related stress**				
Never, rarely, or sometimes	33 480	3487	1 (reference)	1 (reference)
Quite often or almost all the time	8121	1164	1.07 (1.00–1.15)	1.05 (0.99–1.13)[a]
**Work-related hurry**				
Never, rarely, or sometimes	30 435	3356	1 (reference)	1 (reference)
Quite often or almost all the time	10 935	1284	1.08 (1.02–1.16)	1.08 (1.01–1.15)[a]
**Depressive mood**				
Never	26 618	3008	1 (reference)	1 (reference)
Sometimes	21 928	2798	1.08 (1.02–1.14)	1.06 (1.01–1.12)[a]
Often	2658	388	1.17 (1.05–1.30)	1.08 (0.97–1.20)[a]
**Nervousness**				
Never	17 650	2079	1 (reference)	1 (reference)
Sometimes	29 876	3582	1.05 (0.99–1.11)	1.06 (1.01–1.12)[a]
Often	3907	585	1.19 (1.08–1.30)	1.13 (1.03–1.24)[a]
**Exhaustion**				
Never	17 588	1891	1 (reference)	1 (reference)
Sometimes	30 723	3379	1.05 (0.99–1.11)	1.04 (0.99–1.10)[a]
Often	6880	983	1.12 (1.03–1.21)	1.09 (1.01–1.18)[a]

Fully adjusted model adjusted for FINRISK survey year, follow-up time (10-year time slots), age at the end of follow-up (5-year time slots), sex, educational class, body mass index, systolic blood pressure, total cholesterol, smoking, physical activity, and diabetes. [a] No covariate of FINRISK survey year could be included. CI, confidence interval; HR, hazard ratio; IRR, incidence rate ratio.

© 2022 Sulkava S et al. *JAMA Network Open.*

eTable 5. Associations of missing data status with all-cause dementia in basic cause-specific hazard model (Poisson) and subdistribution hazard model (Fine–Gray).

				All-cause dementia	Competing risk of death
				**Cause-specific hazard model (Poisson)**	**Cause-specific hazard model (Poisson)**
	No., all	No., dementia cases	No., competing event	Basic model, IRR (CI 95%)	Basic model, IRR (CI 95%)
**Missing covariates in fully adjusted model, No**	59 707	6789	16 870	1 (reference)	1 (reference)
**Yes**	9521	1418	3460	1.09 (1.02–1.16)	1.20 (1.17–1.23)

Basic model adjusted for follow-up time (10-year time slots), age at the end of follow-up (5-year time slots), FINRISK survey year, sex, and educational class. CI, confidence interval; HR, hazard ratio; IRR, incidence rate ratio.

© 2022 Sulkava S et al. *JAMA Network Open.*

eTable 6. Associations of psychological distress symptoms or non-responsiveness with all-cause dementia in basic cause-specific hazard model (Poisson).

			**Cause-specific hazard model (Poisson)**
Trait	No., all	No., dementia cases	Fully adjusted model, IRR (CI 95%)
**Stress**<br>At the same level as other people	32 399	3107	1 (reference)
More than other people	5760	435	1.23 (1.11-1.36)
No reponse	793	150	1.25 (1.06-1.48)
**Depressive mood**<br>Never	31 676	3726	1 (reference)
Sometimes	26 178	3453	1.13 (1.08-1.18)
Often	3457	512	1.29(1.17-1.41)
No response	1665	335	1.23(1.09-1.38)
**Nervousness**<br>Never	21 372	2627	1 (reference)
Sometimes	35 313	4399	1.07 (1.02-1.12)
Often	4973	743	1.25 (1.15-1.36)
No response	1318	357	1.21 (1.06-1.39)
**Exhaustion**<br>Never	21 471	2428	1 (reference)
Sometimes	37 249	4247	1.07(1.02-1.13)
Often	8868	1259	1.20(1.12-1.29)
No response	1637	273	1.25(1.09-1.42)

Basic model adjusted for follow-up time (10-year time slots), age at the end of follow-up (5-year time slots), FINRISK survey year, sex, and educational class. CI, confidence interval; HR, hazard ratio; IRR, incidence rate ratio.

© 2022 Sulkava S et al. *JAMA Network Open.*

eTable 7. Associations of depressive mood and exhaustion with all-cause dementia in fully adjusted cause-specific hazard model (Poisson) stratified by sex.

	**Men**			**Women**		
Trait	No., all	No., dementia cases	IRR (CI 95%)	No., all	No., dementia cases	IRR (CI 95%)
**Depressive mood** Never	15 726	1464	1 (reference)	12 536	1701	1 (reference)
Sometimes	9834	997	1.12 (1.03–1.21)	13 559	1932	1.07 (1.01–1.15)
Often	1123	117	1.52 (1.26–1.84)	1841	303	1.12 (0.99–1.27)
**Exhausted** Never	10 577	911	1 (reference)	8204	1102	1 (reference)
Sometimes	15 025	1399	1.16 (1.06–1.26)	17 553	2215	1.02 (0.95–1.10)
Often	2965	329	1.32 (1.16–1.51)	4608	718	1.08 (0.98–1.19)

Fully adjusted model adjusted for FINRISK survey year, follow-up time (10-year time slots), age at the end of follow-up (5-year time slots), sex, educational class, body mass index, systolic blood pressure, total cholesterol, smoking, physical activity, and diabetes. CI, confidence interval; IRR, incidence rate ratio.

© 2022 Sulkava S et al. *JAMA Network Open.*

eTable 8. Associations of psychological distress symptoms with all-cause dementia in fully adjusted cause-specific hazard model (Poisson) with covariate of insomnia.

			**Cause-specific hazard model (Poisson)**	**Cause-specific hazard model (Poisson) adjusted for insomnia**
Trait	No., all	No., dementia cases	Fully adjusted model, IRR (CI 95%)	Fully adjusted model, IRR (CI 95%)
**Stress**<br>At the same level as other people	28 626	2463	1 (reference)	1 (reference)
More than other people	5238	363	1.24 (1.11–1.38)	1.17 (1.04–1.31)
**Depressive mood**<br>Never	28 186	3153	1 (reference)	1 (reference)
Sometimes	23 196	2886	1.09 (1.04–1.15)	1.05 (1.02–1.28)
Often	2928	414	1.22 (1.10–1.36)	1.14 (1.02–1.28)
**Nervousness**<br>Never	18 824	2195	1 (reference)	1 (reference)
Sometimes	31 424	3690	1.05 (1.00–1.11)	1.02 (0.97–1.08)
Often	4243	605	1.21 (1.11–1.33)	1.13 (1.02–1.24)
**Exhaustion**<br>Never	18 724	2003	1 (reference)	1 (reference)
Sometimes	32 295	3561	1.08 (1.02–1.14)	1.06 (0.997–1.12)
Often	7483	1031	1.17 (1.08–1.26)	1.11 (1.03–1.21)

Fully adjusted model adjusted for FINRISK survey year, follow-up time (10-year time slots), age at the end of follow-up (5-year time slots), sex, educational class, body mass index, systolic blood pressure, total cholesterol, smoking, physical activity, and diabetes. CI, confidence interval; IRR, incidence rate ratio.

© 2022 Sulkava S et al. *JAMA Network Open.*

eTable 9. Associations of psychological distress symptoms with all-cause dementia in fully adjusted cause-specific hazard model (Poisson) with and without covariate of alcohol consumption in the cohort comprising of the FINRISK Study surveys 1982, 1987 and 1992.

			**Cause-specific hazard model (Poisson)**	**Cause-specific hazard model (Poisson) adjusted for alcohol consumption**
Trait	No., all	No., dementia cases	Fully adjusted model, IRR (CI 95%)	Fully adjusted model and alcohol consumption, IRR (CI 95%)
**Stress**<br>At the same level as other people	29 014	2516	1 (reference)	1 (reference)
More than other people	5278	367	1.23(1.08-1.40)	1.22 (1.06–1.41)
**Depressive mood**<br>Never	28 263	3165	1 (reference)	1 (reference)
Sometimes	23 397	2930	1.03(0.94-1.13)	1.01 (0.92–1.12)
Often	2965	420	1.28(1.05-1.55)	1.30 (1.06–1.60)
**Nervousness**<br>Never	18 910	2207	1 (reference)	1 (reference)
Sometimes	31 682	3746	1.02(0.93-1.12)	1.01 (0.91–1.12)
Often	4290	615	1.39(1.19-1.63)	1.40 (1.18–1.66)
**Exhaustion**<br>Never	18 785	2014	1 (reference)	1 (reference)
Sometimes	32 584	3616	1.04(0.94-1.14)	1.03 (0.93–1.14)
Often	7579	1047	1.24(1.08-1.44)	1.20 (1.03–1.41)

Fully adjusted model adjusted for FINRISK survey year, follow-up time (10-year time slots), age at the end of follow-up (5-year time slots), sex, educational class, body mass index, systolic blood pressure, total cholesterol, smoking, physical activity, and diabetes. CI, confidence interval; IRR, incidence rate ratio.

© 2022 Sulkava S et al. *JAMA Network Open.*

eTable 10. Sensitivity analyses for associations of psychological distress symptoms with Alzheimer's disease in the fully adjusted cause-specific hazard model (Poisson) and subdistribution hazard model (Fine–Gray). (Only individuals with follow-up time ≥ 10 years are included.)

Trait	No., all	No., AD cases	**Cause-specific hazard model (Poisson)** Fully adjusted model, IRR (CI 95%)	**Fine–Gray subdistribution hazard model** Fully adjusted model, HR (CI 95%)
**Stress**				
At the same level as other people	27 292	1852	1 (reference)	1 (reference)
More than other people	4931	234	1.07 (0.93–1.22)	1.00 (0.87-1.14)[a]
**Depressive mood**				
Never	26 618	2312	1 (reference)	1 (reference)
Sometimes	21 928	2058	1.02 (0.95–1.08)	1.00 (0.94–1.07)
Often	2658	260	1.05 (0.92–1.20)	0.97 (0.85–1.11)
**Nervousness**				
Never	17 650	1559	1 (reference)	1 (reference)
Sometimes	29 876	2709	1.04 (0.98–1.11)	1.06 (0.996–1.14)
Often	3907	399	1.09 (0.97–1.23)	1.05 (0.94–1.18)
**Exhausted**				
Never	17 588	1468	1 (reference)	1 (reference)
Sometimes	30 723	2525	1.02 (0.95–1.08)	1.00 (0.94–1.07)
Often	6880	683	1.06 (0.97–1.17)	1.00 (0.91–1.09)

Fully adjusted model adjusted for FINRISK survey year, follow-up time (10-year time slots), age at the end of follow-up (5-year time slots), sex, educational class, body mass index, systolic blood pressure, total cholesterol, smoking, physical activity, and diabetes. [a] No covariate of FINRISK survey year could be included. AD, Alzheimer's disease; CI, confidence interval; HR, hazard ratio; IRR, incidence rate ratio.

© 2022 Sulkava S et al. *JAMA Network Open.*

eTable 11. Depressive mood and self-reported diagnosis of depression stratified by sex in the FINRISK 2002 survey.

	Men	Women
**Trait**	**Self-reported diagnosis of depression, No. (%)**	**Self-reported diagnosis of depression, No. (%)**
**Depressive mood**		
Never	24/2611 (0.9)	39/2551 (1.5)
Sometimes	112/1154 (9.7)	185/1760 (10.5)
Often	102/154 (66.2)	138/249 (55.4)

© 2022 Sulkava S et al. *JAMA Network Open.*

eMethods

Description of all statistical tests

To examine association of psychological distress with dementia, we used in parallel two methods accounting for the competing risk of death, cause-specific hazard model and subdistribution hazard model. As a cause-specific hazard model we used Poisson model and as a subdistribution hazard model Fine-Gray model. Sensitivity analyses for reverse-causation excluded individuals with follow-up time < 10 years. The main analyses were shown for basic model with the most essential covariates and for the fully adjusted model.

As explanatory secondary analyses we performed cause-specific hazard model analyses stratified by baseline age (< 45 years; 45–65 years; > 65 years). Interaction terms with sex in the cause-specific hazard model analyses were examined, and when significant, analyses stratified for sex were performed. In addition, cause-specific hazard models with covariates of insomnia and alcohol consumption were studied. For AD, we performed, similarly to all-cause dementia, in parallel cause-specific hazard model and subdistribution hazard model. Association of the missing data status with dementia in the fully adjusted model was studied in cause-specific hazard model and subdistribution hazard model. In addition, non-responsiveness to psychological distress variable was coded as a separate category, and cause-specific hazard model analysis for dementia was performed. To study differences in the baseline values of the exposures and covariates between those with missing data on the fully adjusted model and those with nonmissing data, we used Pearson's chi-square test for categorical variables and one-way analysis of variance for continuous variables. Correlation of the exposure traits was examined with Spearman's correlation coefficient. Here, the significance threshold was P<.01 The significance threshold of P<.05 or confidence intervals of 95 % were used throughout the study, except for Spearman's correlation.

Study population

Ethnicity was not ascertained in the National FINRISK study surveys 1972-2007. It is also not collected in the Finnish registers.

Outcome

For diagnoses of all-cause dementia, and secondary outcome Alzheimer's disease (AD), we used combined information from the Finnish national registers: the Hospital Discharge Register and the Causes of Death Register. An individual was coded as having all-cause dementia if fulfilling the criteria for AD or if having the diagnosis of dementia or specific dementia disorder in the Causes of Death Register or the Hospital Discharge Register. For AD the specific diagnosis codes included were: years 1987–1995 ICD-9 (International Classification of Diseases): 3310; year 1996 onward ICD-10: F00, G30. For all-cause dementia the diagnosis codes were: years 1972–1986 ICD-8: 290; years 1987–1995 ICD-9: 3310, 4378A, 290; year 1996 onward ICD-10: F00, F01, F02, F03, G30. In addition, we used the Drug Reimbursement Register (reimbursement for AD medication, code 307), and the Social Insurance Institution's information

© 2022 Sulkava S et al. *JAMA Network Open.*

from prescribed and bought dementia drugs (1995 onward, considered as a case if received ATC class N06D drugs: donepezil, rivastigmine, memantine, and galantamine). Date information accompanied the events. In the analysis of AD, individuals with non-AD dementia were treated as censored

The Hospital Discharge Register, maintained by the Finnish Institute for Health and Welfare, receives information about inpatient stays in Finnish public hospitals and, since 1998, outpatient visits. The information on the Causes of Death Register was complete because the register includes deaths of Finnish citizens in Finland and abroad [1]. Different sources for diagnoses are separated in eTable 1. The most common source of the earliest diagnosis was the Drug Reimbursement Register for both all-cause dementia and AD.

Accuracy of the all-cause dementia and Alzheimer's disease (AD) diagnoses based on the Finnish registers is good (96% and 100% positive predictive value) when comparing with clinical diagnostic examination, but some underestimation of occurrence may occur because, when used in combination, the Hospital Discharge Register and the Drug Reimbursement Register led to a sensitivity of 71% for AD [2]. The information from the Causes of Death Register and purchased medication was not, however, used in their definition of set diagnosis as we do; thus the sensitivity is likely to be higher in our study in the later years. However, before the 1990s the rarity of AD diagnostics as well as a lack of AD medication (first described in 1996, eMethods) has surely made the sensitivity lower than that in the later years.

The first diagnoses for AD in the data set appeared in year 1987. Before 1987 and partly after that the cases of AD are likely to be classified as cases of dementia in the Causes of Death Register or in the Hospital Discharge Register. The inaccuracy in the diagnosis of AD in the early years may weaken the associations, because the follow-up for the first FINRISK survey began in 1972 in our study.

The drug-related registers, which are the main source of diagnosis (eTable 1), begin to have information on AD medication only after 1996. After the beginning of the systematic use of dementia drugs, the Drug Reimbursement Register is likely to be a relatively reliable source for the diagnosis of AD, because to receive reimbursement the patient has to be diagnosed for AD by a physician specialized in neurology or geriatrics. In Finland, examination typically includes a medical interview and a clinical examination, brain imaging by magnetic resonance imaging, measures for cognitive decline like those of the Consortium to Establish a Registry for Alzheimer's Disease (CERAD), and laboratory measurements for differential diagnostics [3]. From year 2012, donepezil was not part of the special reimbursement and, from year 2013, neither was memantine. After these changes, the diagnosis based on AD medication was likely to lose some specificity when examination by a doctor specialized in neurology or geriatrics was no longer needed for reimbursement. However, after that, most patients still visit a specialized doctor for diagnosis in Finland, and medication purchases are likely to reflect AD relatively well, because only rarely is the AD medication used for other types of dementias like Lewy body disease or vascular dementia.

© 2022 Sulkava S et al. *JAMA Network Open.*

Additional Covariates

As an additional covariate we used alcohol consumption, which was dichotomized based on previous evidence showing increased risk of dementia associated with alcohol consumption at a level of > 14 units per week [4]. The question assessed alcohol units consumed during the last week, covering the consumption of beer, long drinks, spirits, and wine. The question was available for the FINRISK Study surveys 1982, 1987, and 1992.

Additional results

When comparing men and women with depressive mood "often" in the FINRISK survey 2002 with self-reported diagnosed depression questioned, we observed more depression among men (102/154, 66.2% vs 138/249, 55.4%; Pearson's R =-0.107, *P* = .032, df = 402, eTable 11 in the Supplement).

References for eMethods

1. Jousilahti P, Salomaa V, Kuulasmaa K, Niemelä M, Vartiainen E. Total and cause specific mortality among participants and non-participants of population based health surveys: A comprehensive follow up of 54 372 finnish men and women. *J Epidemiol Community Health*. 2005;59(4):310-315. doi: 59/4/310 [pii].

2. Solomon A, Ngandu T, Soininen H, Hallikainen MM, Kivipelto M, Laatikainen T. Validity of dementia and alzheimer's disease diagnoses in finnish national registers. *Alzheimers Dement*. 2014;10(3):303-309. doi: 10.1016/j.jalz.2013.03.004 [doi].

3. Update on current care guideline: Memory disorders. *Duodecim*. 2017;133(8):756-757.

4. Sabia S, Fayosse A, Dumurgier J, et al. Alcohol consumption and risk of dementia: 23 year follow-up of whitehall II cohort study. *BMJ*. 2018;362:k2927. doi: 10.1136/bmj.k2927 [doi].

© 2022 Sulkava S et al. *JAMA Network Open.*
