# Supplementary material for: Association Between Psychological Distress and Incident Dementia in a Population-Based Cohort in Finland
Source: JAMA Netw Open. 2022 Dec 15;5(12):e2247115. doi: 10.1001/jamanetworkopen.2022.47115 (PMC9856411; doi:10.1001/jamanetworkopen.2022.47115)
Supplement: Supplement 2. — Data Sharing Statement [file jamanetwopen-e2247115-s002.pdf]

Data Sharing Statement

Sulkava. Association Between Psychological Distress and Incident Dementia in a Population-Based Cohort in Finland. *JAMA Netw Open*. Published December 15, 2022.
doi:10.1001/jamanetworkopen.2022.47115

Data
Data available: No

Additional Information
Explanation for why data not available: Access to the National FINRISK study data can be applied through the THL Biopank.
